# Strict bladder filling and rectal emptying during prostate SBRT: Does it make a dosimetric or clinical difference?

**DOI:** 10.1186/s13014-020-01681-6

**Published:** 2020-10-16

**Authors:** David J. Byun, Daniel J. Gorovets, Lauren M. Jacobs, Laura Happersett, Pengpeng Zhang, Xin Pei, Sarah Burleson, Zhigang Zhang, Margie Hunt, Sean McBride, Marisa A. Kollmeier, Michael J. Zelefsky

**Affiliations:** 1 Department of Radiation Oncology, NYU Langone Health, 160 East 34th St, New York, NY USA; 2 Department of Radiation Oncology, Memorial Sloan Kettering Cancer Center, 1275 York Ave, New York, NY 10065 USA; 3 Department of Medical Physics, Memorial Sloan Kettering Cancer Center, 1275 York Ave, New York, NY USA; 4 Department of Epidemiology and Biostatistics, Memorial Sloan Kettering Cancer Center, 1275 York Ave, New York, NY USA

**Keywords:** SBRT, Prostate cancer, Bladder volume, Rectum volume, Interfractional organ displacement

## Abstract

**Background:**

To evaluate inter-fractional variations in bladder and rectum during prostate stereotactic body radiation therapy (SBRT) and determine dosimetric and clinical consequences.

**Methods:**

Eighty-five patients with 510 computed tomography (CT) images were analyzed. Median prescription dose was 40 Gy in 5 fractions. Patients were instructed to maintain a full bladder and empty rectum prior to simulation and each treatment. A single reviewer delineated organs at risk (OARs) on the simulation (Sim-CT) and Cone Beam CTs (CBCT) for analyses.

**Results:**

Bladder and rectum volume reductions were observed throughout the course of SBRT, with largest mean reductions of 86.9 mL (19.0%) for bladder and 6.4 mL (8.7%) for rectum noted at fraction #5 compared to Sim-CT (*P* < 0.01). Higher initial Sim-CT bladder volumes were predictive for greater reduction in absolute bladder volume during treatment (ρ = − 0.69; *P* < 0.01). Over the course of SBRT, there was a small but significant increase in bladder mean dose (+ 4.5 ± 12.8%; *P* < 0.01) but no significant change in the D2cc (+ 0.8 ± 4.0%; *P* = 0.28). The mean bladder trigone displacement was in the anterior direction (+ 4.02 ± 6.59 mm) with a corresponding decrease in mean trigone dose (− 3.6 ± 9.6%; *P* < 0.01) and D2cc (− 6.2 ± 15.6%; *P* < 0.01). There was a small but significant increase in mean rectal dose (+ 7.0 ± 12.9%, *P* < 0.01) but a decrease in rectal D2cc (− 2.2 ± 10.1%; *P* = 0.04). No significant correlations were found between relative bladder volume changes, bladder trigone displacements, or rectum volume changes with rates of genitourinary or rectal toxicities.

**Conclusions:**

Despite smaller than expected bladder and rectal volumes at the time of treatment compared to the planning scans, dosimetric impact was minimal and not predictive of detrimental clinical outcomes. These results cast doubt on the need for excessively strict bladder filling and rectal emptying protocols in the context of image guided prostate SBRT and prospective studies are needed to determine its necessity.

## Background

Stereotactic body radiation therapy (SBRT) is gaining momentum for its clinical application of the treatment of localized prostate cancer. The use of SBRT is becoming a viable alternative with preliminary clinical trials and single institutions reporting equal or superior biochemical recurrence rates to the traditional forms of external beam radiation therapy (EBRT) and radical prostatectomy [1–11]. However, the accuracy of radiation is more critical for SBRT in comparison to conventional EBRT given its higher dose per fraction, minimal margin for error in target volume localization, and sensitivity of late responding normal tissue to hypofractionation [12]. Thus far, reports of toxicity outcomes after SBRT have been encouraging. Comparable rates of genitourinary and bowel toxicities to IMRT have been reported in preliminary trials and multi-institutional experiences [1, 4–6, 13]. Recently, a Phase III non-inferiority clinical trial comparing conventional prostate EBRT to a seven-fraction prostate SBRT regimen (4270 cGy) revealed a comparable acute end-of-treatment urinary and bowel toxicity outcomes, as well as at 5-year follow-up [7].

We hypothesize that variations in bladder and rectum filling during the course of prostate SBRT could result in unexpected doses being delivered to critical normal tissues and possibly affect the rate of treatment-related toxicity. To our knowledge, an evaluation of anatomic variations in the bladder and rectal filling in an SBRT prostate cancer cohort has yet to be conducted. To this end, we sought to determine the amount of interfractional pelvic organ volume changes and displacement during prostate SBRT, as well as the potential dosimetric and clinical impact on these organs at risk (OARs) in a cohort of patients who were placed on a strict pre-treatment bladder filling and rectal emptying pre-treatment regimen.

## Methods

### Patient population, simulation procedures and target volumes

Following Institutional Review Board approval, data were obtained from medical records of 85 consecutive patients treated with 5-fraction SBRT to the prostate from September 2014 to August 2015 at our institution. Each patient underwent a 2–3 mm slice thickness simulation computed tomography (Sim-CT) in the supine position with thermoplast immobilization (Aquaplast; Qfix, Avondale, PA). To maintain an adequately filled bladder for simulation and treatment, patients were instructed to drink 2 cups of water 1 h prior to each session. Strict rectal protocol of fiber supplementation, simeticone, and rectal enemas 3 h prior to simulation and each treatment was advised. During the course of this study, rectal spacers were not used in this patient cohort. Based on the simulation CT and fusion of available pre-treatment prostate MRI, clinical target volume (CTV) was defined as prostate gland plus proximal ≥ 1 cm of the seminal vesicles. The planning target volume (PTV) was defined as CTV expansion of 5 mm throughout except for 3 mm posterior.

### Organ displacement management and delineation

To adjust for any inter-fractional clinical target volume (CTV) shift, kV orthogonal radiographs and Cone Beam CT (CBCT) images were acquired prior to each treatment to realign intraprostatic fiducial markers to the planned CTV position that had been determined at the Sim-CT. Treating physicians visually assessed the adequacy of bladder filling and rectal volumes on the CBCTs compared to initial Sim-CT prior to treatment delivery.

Using the Eclipse^®^ Treatment Planning System (Varian Medical Systems; Palo Alto, CA), CBCTs were registered and blended with the Sim-CT for each patient. A single reviewer determined anatomical delineations of the CTV (prostate and seminal vesicles), bladder wall, bladder trigone, and rectum on the baseline Sim-CT, as well as the 5 blended CBCTs, for each patient (n = 510 total images). For each scan, the rectum was contoured from the rectosigmoid flexure superiorly to the ischial tuberosity inferiorly. Consistency of rectal length was maintained through all 6 sets of CT images for each patient. Bladder trigone was defined as the triangular posteroinferior region of the bladder wall extending from the ureteral orifices superiorly to the internal urethral sphincter inferiorly.

### Volume, position, and dosimetric evaluation

Target and OAR volumes were obtained from the treatment planning system. Interfractional organ displacements were quantified by calculating the organ center of mass (COM) differences on the individual pre-treatment CBCTs aligned to CTV and intraprostatic fiducial markers in relation to the baseline Sim-CTs. To account for the reproducibility of the pelvic organ delineation by the reviewer, 10 randomly selected CBCTs were re-contoured at the end of data collection. Intraclass correlation coefficient (ICC) was used to evaluate the level of reproducibility in the organ center of mass displacements and organ volumes, where a coefficient of 1 implies perfect reproducibility and 0 implies no consistency in delineation by the single reviewer [14].

CBCTs were fused to Sim-CT and fractional doses for the newly delineated CBCT structures were recalculated based on the original treatment plan. To account for any variation in dose prescriptions and OAR dimensional variations at the level of the CTV, fractional mean OAR doses and fractional maximum doses to 2 cc of OAR (D2cc) were represented as a percentage of the initial dose at simulation.

### Statistical analysis

A two-tailed Wilcoxon signed-rank test was used to evaluate differences between volume, displacement, mean doses, and D2cc of pelvic organs at Sim-CT compared to subsequent fractionations. Absolute inter-fraction volume change was defined as the volume at fraction #5 subtracted from the volume at Sim-CT (denoted V_5-Sim_). Simple regression analysis (denoted r^2^) was used as a proxy to represent the degree of variance of intra-patient volume change through the course of treatment. Spearman’s rank order correlation (ρ) was used to assess if pre-treatment International Prostate Symptom Score (IPSS) was predictive of bladder volume changes, and whether bladder and rectum volumes at Sim-CT was predictive of volume change, organ displacement, or dose variation.

A two-sided Wilcoxon rank sum test was implemented to evaluate whether relative bladder volume, rectum volume, and bladder trigone displacement variations were predictive of acute (< 30 days from completion of treatment) and late (≥ 30 days from completion of treatment) genitourinary (GU) or gastrointestinal (GI) toxicities. GU/GI toxicities were defined based on the Common Terminology Criteria for Adverse Events version 5.0 and categorized as a clinically significant toxicity event if grade 2 or higher.

The significance level at which a null hypothesis would be rejected was defined as *P* < 0.05. Statistical analyses were conducted using SPSS^®^ software Version 23.0 (IBM Corp.; Armonk, NY).

## Results

### Patient characteristics

Of the 85 total patients, 61 received prescription doses of 40 Gy in 5 fractions, 10 received prescription doses of 37.5 Gy in 5 fractions, and 14 received combination therapy of low dose rate brachytherapy in addition to 5-fraction SBRT to prescription doses of 25 Gy. The mean age was 69.7 years old (range 51–85 years), with tumor stages ranging from T_1–3_N_0_M_0_ (n = 79) to T_1–4_N_0–1_M_0–1_ (n = 6). The median follow-up was 2.85 years.

### Reproducibility of organ delineation

Comparisons of the initial pelvic organ delineation in 10 randomly chosen CBCTs and the repeat delineations by a single reviewer showed excellent reproducibility in COM displacement as well as organ volume (ICC coefficient range 0.93–1.00).

### Bladder and rectum volume

**Fig. 1:**
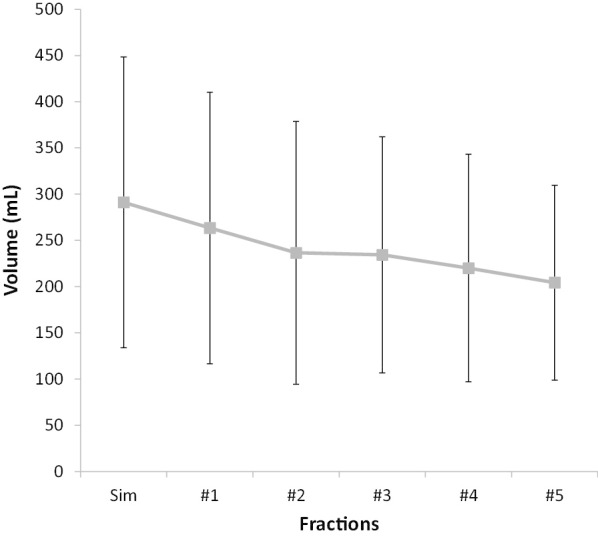
Trends in bladder volume from simulation CT (Sim) to fraction #5. Error bars represent standard deviation. Bladder volume from Sim-CT to fraction #5 decreased by 19.9% (Wilcoxon signed-rank test: *P* < 0.01)

**Fig. 2:**
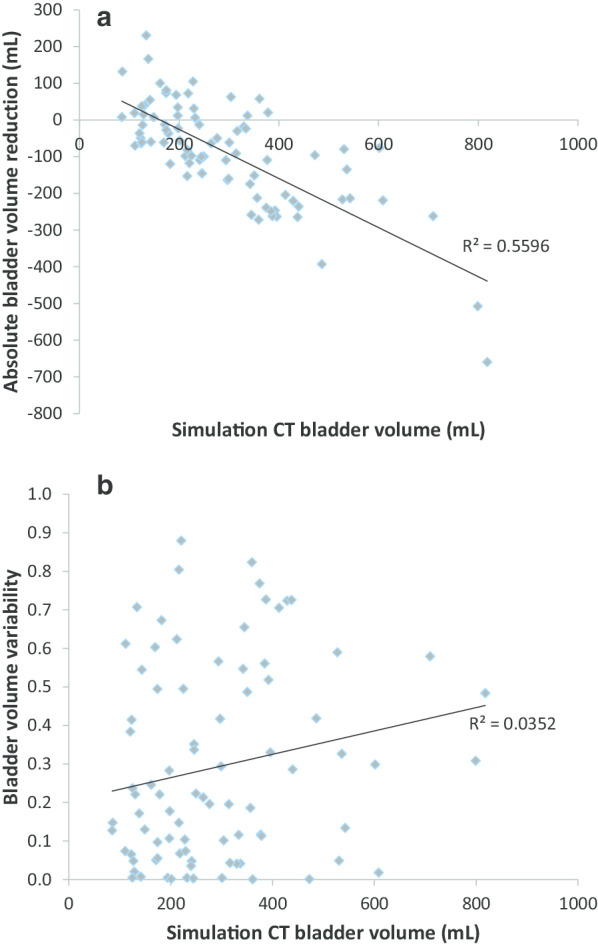
**a** Sim CT volume in relation to absolute volume reduction (V_5-sim_). The goodness of fit of the linear regression model is represented by R^2^ (Spearman's correlation = − 0.69; *P* < 0.001). **b** Sim CT volume in relation to bladder volume variability (r^2^) in individual patients through 5 fractions. The goodness of fit of the linear regression model is represented by R^2^ (Spearman's correlation = 0.18; *P* = 0.09)

The mean bladder volume at the time of the baseline Sim-CT was 291.2 ± 157.3 mL. Bladder volumes in the subsequent CBCTs were as follows: 263.4 ± 147.1 mL at fraction #1, 236.6 ± 145.0 mL at #2, 234.3 ± 128.1 mL at #3, 220.0 ± 127.6 mL at #4, and 204.3 ± 106.9 mL at #5 (Fig. 1). The average bladder volume reduction per patient from Sim-CT to fraction #5 (V_5-Sim_Bladder) significantly decreased by 86.9 mL or 19.0% (Wilcoxon signed-rank test: *P* < 0.01). In 10 patients, exact bladder volume could not be delineated due to limitations in field of view in CBCTs compared to full pelvic simulation CTs. After accounting for these limitations and excluding patients with sub-optimal CBCTs, V_5-Sim_Bladder significantly decreased by 90.2 mL or 20.6% (Wilcoxon signed-rank test: *P* < 0.01). Sim-CT bladder volume was inversely correlated with V_5-Sim_Bladder (ρ = − 0.69; *P* < 0.01); thus, larger bladder volume at simulation was predictive of larger bladder volume loss toward fraction #5 (Fig. 2a). However, pre-treatment IPSS score, a metric for urinary dysfunction, was not predictive of relative bladder volume reduction through treatment (ρ = − 0.07; *P* = 0.5). Sim-CT bladder volume was not a significant predictor of variance in intra-patient bladder volume change (r^2^) (Spearman's correlation = 0.18; *P* = 0.09) (Fig. 2b).

**Fig. 3:**
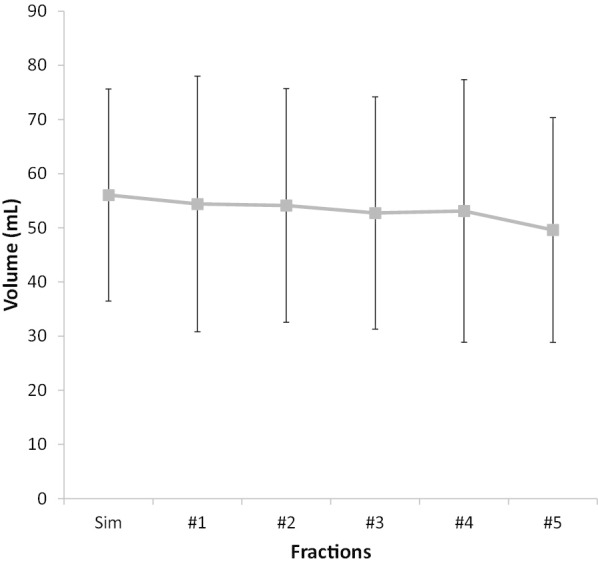
Trends in rectum volume from simulation CT (Sim) to fraction #5. Error bars represent standard deviation. Rectum volume from Sim-CT to fraction #5 decreased by 8.7% (Wilcoxon signed-rank test: *P* < 0.01)

**Fig. 4:**
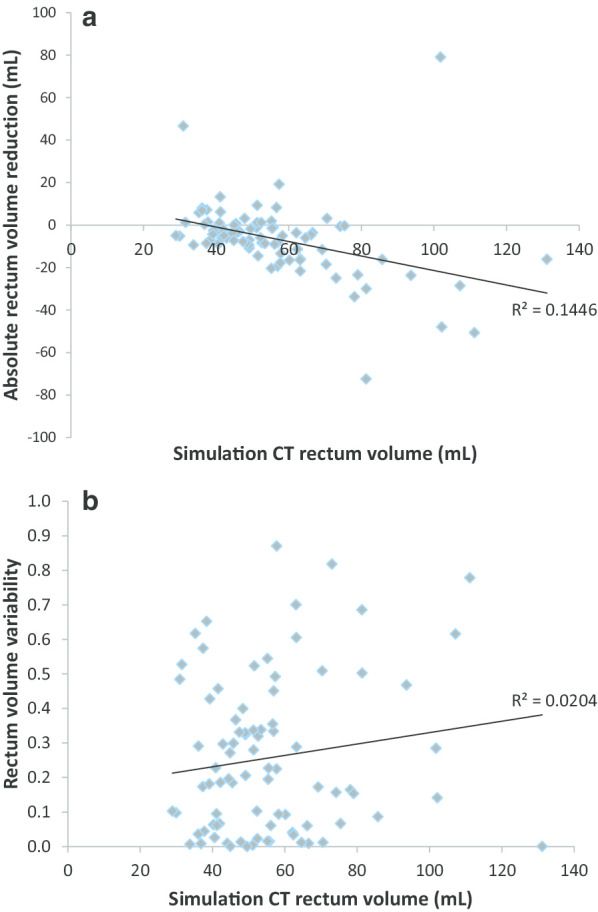
**a** Sim CT volume in relation to absolute rectum reduction (V_5-sim_). The goodness of fit of the linear regression model is represented by R^2^ (Spearman's correlation = − 0.51; *P* < 0.01). **b** Sim CT volume in relation to rectum volume variability (r^2^) in individual patient through 5 fractions. The goodness of fit of the linear regression model is represented by R^2^ (Spearman's correlation = 0.08; *P* = 0.48)

Baseline Sim-CT rectum volume was 56.1 ± 19.6 mL, with subsequent rectum volumes measuring 54.4 ± 23.6 mL at fraction #1, 54.1 ± 21.7 mL at #2, 52.7 ± 21.5 mL at #3, 53.1 ± 24.2 mL at #4, and 49.6 ± 20.7 mL at #5 (Fig. 3). Rectum volume significantly decreased by 6.4 mL or 8.7% from Sim-CT to fraction #5 (V_5-Sim_Rectum) (Wilcoxon signed-rank test: *P* < 0.01). A weak but significant association between the Sim-CT rectum volume and the V_5-Sim_Rectum was observed (Spearman's correlation = − 0.51; *P* < 0.01); however, Sim-CT rectum volume was not a predictor of rectum r^2^ (Spearman's correlation = 0.08; *P* = 0.48 (Fig. 4a, b).

### Pelvic organ displacement

Patients were positioned for treatment based on intraprostatic fiducial marker matching, therefore the prostate CTV COM had minimal inter-fractional displacements and typically remained within our PTV margins (lateral: − 0.11 ± 0.82 mm; anterior/posterior: + 0.64 ± 1.75 mm; superior/inferior: + 0.07 ± 1.37 mm). However, OAR displacements were more pronounced (Table 1). Increased inferior bladder (all fractions: − 2.82 ± 8.98 mm, fraction 5: − 3.40 ± 8.84 mm), anterior bladder trigone (all fractions: + 2.64 ± 6.25 mm; fraction 5: + 4.02 ± 6.59 mm), and superior rectum (all fractions: + 3.19 ± 5.84 mm; fraction 5: + 2.38 ± 5.74 mm) displacements were observed during all fractions compared to the initial COM at Sim-CT. Further, the percentages of inter-fractional displacements surpassing 3 mm were considerable in the inferior bladder (all fractions: 75% > 3 mm; fraction 5: 82% > 3 mm), anterior bladder trigone (all fractions: 53% > 3 mm; fraction 5: 52% > 3 mm), and superior rectum displacements (all fractions: 62% > 3 mm; fraction 5: 65% > 3 mm).

**Table 1:** Displacement of pelvic organ center of mass compared to simulation CT (in mm)

	All fractions		Fraction 5	
	Mean ± SD	> 3mm (%)^a^	Mean ± SD	> 3mm (%)^a^
CTV^b^				
Lateral	− 0.11 ± 0.82	1	− 0.07 ± 0.87	1
Anterior/posterior	0.64 ± 1.75	10	0.66 ± 1.73	12
Superior/inferior	0.07 ± 1.37	3	0.37 ± 1.44	6
Bladder				
Lateral	− 0.03 ± 2.84	26	0.02 ± 2.91	27
Anterior/posterior	− 0.70 ± 6.45	60	− 0.86 ± 6.24	62
Superior/inferior	− 2.82 ± 8.98	75	− 3.40 ± 8.84	82
Bladder Trigone				
Lateral	− 0.19 ± 1.64	8	− 0.14 ± 1.90	12
Anterior/posterior	2.64 ± 6.25	53	4.02 ± 6.59	52
Superior/inferior	− 0.50 ± 3.43	8	− 0.55 ± 3.29	6
Rectum				
Lateral	− 0.13 ± 1.86	9	− 0.12 ± 1.87	8
Anterior/posterior	− 0.21 ± 3.28	26	0.05 ± 3.14	24
Superior/inferior	3.19 ± 5.84	62	2.38 ± 5.74	65

Positive values denote superior, anterior, or leftward displacement of organ center of mass

^a^> 3 mm refers to percentage of > 3 mm displacements relative to center of mass at simulation CT

^b^CTV denotes prostate and seminal vesicles

### Pelvic organ dosimetry

Over the course of SBRT, there was a significant but small increase in bladder mean dose (all fractions: + 3.7 ± 13.6%; fraction 5: + 4.5 ± 12.8%; *P* < 0.01), a decrease in bladder trigone mean dose (all fractions: − 2.1 ± 11.0%; fraction 5: − 3.6 ± 9.6%, *P* < 0.01), and an increase in rectum mean dose (all fractions: + 8.4 ± 14.3%; fraction 5: + 7.0 ± 12.9%, *P* < 0.01) compared to baseline Sim-CT (Table 2). Evaluating for maximum dose exposure to OARs, there was no significant change in bladder D2cc dose (all fractions: + 0.4 ± 2.7%; fraction 5: + 0.8 ± 4.0%; *P* = 0.28), a small but significant decrease in bladder trigone D2cc dose (all fractions: − 3.0 ± 23.9%; fraction 5: − 6.2 ± 15.6%; *P* < 0.01), and a small but significant decrease in rectum D2cc dose (all fractions: − 1.0 ± 10.0%; fraction 5: − 2.2 ± 10.1%; *P* = 0.04) from simulation D2cc.

**Table 2:** Trends in mean dose from simulation CT to fraction #5

	Prostate	Bladder	Bladder Trigone	Rectum
	Mean ± SD	Mean ± SD	Mean ± SD	Mean ± SD
Simulation	102.80% ± 1.00%	42.10% ± 18.30%	93.50% ± 9.20%	43.90% ± 6.40%
Fraction 1	102.50% ± 1.00%	44.50% ± 18.80%	92.50% ± 9.90%	47.90% ± 8.70%
Fraction 2	103.40% ± 8.10%	46.80% ± 20.80%	92.10% ± 9.60%	47.50% ± 7.70%
Fraction 3	102.50% ± 1.00%	44.80% ± 19.80%	90.90% ± 10.80%	47.20% ± 8.30%
Fraction 4	102.50% ± 1.00%	46.40% ± 20.50%	90.20% ± 10.10%	47.10% ± 7.30%
Fraction 5	102.10% ± 3.20%	46.70% ± 19.90%	89.80% ± 10.50%	46.60% ± 7.10%
Δdose (%)	− 0.60% ± 2.90%	4.50% ± 12.80%	− 3.60% ± 9.60%	7.00% ± 12.90%
Δdose (Gy)	− 0.2 ± 1.1	1.8 ± 4.8	− 1.5 ± 3.3	0.96 ± 1.87

ΔDose denotes percentage or absolute mean dose change from simulation CT to fraction #5. Fraction #1–5 mean doses were significantly different from simulation dose in all OARs (all *P* < 0.01)

### Volume as a predictor of displacement and dose

A significant correlation was found between V_5-Sim_Bladder (bladder volume reduction over the course of treatment) and anterior displacement of the bladder trigone (ρ = − 0.50; *P* < 0.001). Anterior bladder trigone displacement was also significantly associated with Sim-CT bladder volume (ρ = 0.30; *P* = 0.005) and r^2^ (ρ = 0.31; *P* = 0.004). Despite these findings, bladder trigone dosimetry was not significantly associated with V_5-Sim_Bladder (*P* = 0.12), V_Sim-CT_Bladder (*P* = 0.35), or r^2^ (*P* = 0.94). V_5-Sim_Rectum (rectal volume reduction over the course of treatment) was associated with superior rectal displacement (ρ = 0.27; *P* = 0.01) and mean rectal dose change (ρ = 0.27; *P* = 0.01). Rectal volume variation and volume at Sim-CT were not significantly associated with rectal displacement (*P* = 0.89; *P* = 0.92, respectively) or dosimetry (*P* = 0.61; *P* = 0.98, respectively).

### Organ volume and position as predictors of treatment-related sequalae

In 71 patient who underwent single modality SBRT, clinically significant acute GU toxicity (grade 2) were observed in 12.7%, late grade 2 GU toxicity in 35.2% of patients (Table 3). No clinically significant acute GI toxicity events were seen and only 4.3% of patients presented with late grade 2 GI toxicity. No significant correlations were found between relative bladder volume changes, rectum volume changes, or anterior bladder trigone displacements with rate of GU/GI toxicities in 71 patients who underwent single modality SBRT alone. Relative bladder volume changes from simulation to fraction 5 were not predictive of acute GU toxicity events (*P* = 0.47) or late GU toxicity events (*P* = 0.28). Anterior bladder trigone displacement was also not significantly associated with an increase in acute GU toxicity (*P* = 0.27) or late GU toxicity (*P* = 0.39). Due to the lack of acute GI toxicity events (n = 0) and insufficient late GI toxicity events (n = 3; 4.3%), data were not sufficient for statistical analysis. Patients who received combination SBRT and brachytherapy (n = 14) were excluded from toxicity analysis to account for the possible confounding effect of toxicities related to low dose rate seed placement.

**Table 3:** Acute and late genitourinary and gastrointestinal toxicity events in single modality prostate SBRT cases (N = 71)

	Genitourinary toxicities		Gastrointestinal toxicities	
	< Grade 2 (%)	Grade 2 (%)	< Grade 2 (%)	Grade 2 (%)
Acute (< 30 days)	12 (16.9)	9 (12.7)	2 (2.8)	0 (0.0)
Late (≥ 30 days)	16 (22.5)	25 (35.2)	11 (15.5)	3 (4.3)

## Discussion

Optimal bladder and rectal filling for external beam radiation therapy for prostate cancer is a key topic of interest [15–17]. Full bladder protocols have the potential to reduce OAR dose exposure via displacement of the small bowel and bladder away from the target volume, with the caveat of increased volume variability [18–20]. Similarly, smaller rectal volumes can reduce dose exposure, but are more difficult to reproduce. Although we could infer from the findings of conventional EBRT cohort evaluation, to our knowledge, there has been no prior evaluation or report of inter-fraction OAR displacement and the dosimetric implications in patients undergoing image-guided prostate SBRT.

Despite instructions to maintain a consistently full bladder at simulation and during treatment, we observed volume variability and a systematic decline in bladder volume through the course of therapy with the largest reduction at the 5th fraction. We hypothesize that this could be related to either radiation-related bladder capacity changes due to acute inflammation, increased efficiency of patient setup, and/or diminished adherence to the filling protocol as the treatment course progressed. Within the cohort analyzed, pre-treatment urinary dysfunction reflected by IPSS score was not associated with decline in bladder volume during treatment.

Several past evaluations of patients treated with conventionally fractionated EBRT with full bladder protocols have reported similar results [15, 21, 22]. For instance, a prospective study by Nakamura et al. [17] showed a significant decline of 38% in bladder volume from fraction #1 to #30. To develop a feedback mechanism for consistent inter-fraction bladder volumes, Stam et al. [22] used daily bladder scans to provide patients with verbal feedback on fluid consumption to improve bladder filling. While there was a moderate improvement in volume reduction in the bladder scan group (19% decline) compared to the control group (31%), the authors concluded that the difference was not sufficient to deem their technique clinically applicable. In a series of 41 patients undergoing conventionally fractionated EBRT, O’Doherty et al. [23] found that using written instructions resulted in more consistent bladder filling throughout treatment, with an association between patient’s subjective bladder fullness and actual bladder volume. Thus, written instructions may help patients maintain a moderately consistent bladder volume for prostate SBRT as well.

Our results, however, call into question the need for such strict bladder filling instructions when using modern radiotherapy techniques that employ more accurate MRI-based target delineation and smaller PTVs facilitated by advanced inter- and intra-fraction image guidance. While significant, it is uncertain if our observation of increased mean dose exposure to the bladder with a decline in bladder filling would ultimately translate to clinically significant GU sequelae in a prospective setting. Our retrospective assessment of the available acute and late GI and GU toxicities data certainly did not reveal a clear correlation to bladder volume and displacement variations.

To date, there have been relatively few defined dose-volume relationships for prostate SBRT that have predicted greater significant GU toxicity. Repka et al. [24] found bladder wall D15.5% > 32.6 Gy to be significantly associated with acute urinary toxicity. Therefore, the volume of the bladder receiving high doses might be more relevant than the mean dose. In the current study, bladder D2cc did not change significantly over the course of treatment, suggesting that the area of bladder exposed to high doses was similar to what we expected based on the DVHs generated during treatment planning. Additionally, anatomical sub-sites of the bladder or urethra (i.e., bladder trigone/neck or membranous urethra) could be relatively more important to avoid with high doses of radiation [25, 26]. Analysis of the bladder trigone was of specific interest given our previously published analyses of patients treated with conventionally fractionated EBRT and low-dose rate brachytherapy, which revealed a strong association between urinary toxicity and exposure to the bladder neck region [25, 26]. Overall bladder volume reduction over the course of SBRT observed in the current study was accompanied by a displacement of the trigone anteriorly and away from areas of highest dose exposure. This phenomenon may be explained by collapsing of the overextended bladder walls in the axial plane with a reduction in urine volume between fractions, as illustrated by the inferior shift in the center of mass of the bladder from simulation to the end of treatment. Anterior displacement of the bladder trigone may therefore represent a surrogate for changes in the surface area of the posterior bladder wall. While previous studies regarding bladder filling emphasized the importance of reproducibility of a full bladder to reduce urinary toxicity, the bladder volume reduction observed here and increased mean bladder dose may minimally contribute to toxicity if the D2cc remains unchanged and the trigone is simultaneously spared potentially deleterious dose exposure [27, 28]. Clearly further exploration of the optimal bladder filling for simulation and treatment in the setting of a highly precise treatment, such as prostate SBRT, is needed.

Although not as substantial as bladder volume reductions, rectal volume also showed a decreasing trend over the course of the 5 fraction SBRT, with a significant but small increase in mean dose exposure to the rectal wall but decrease in D2cc. These findings are corroborated by studies in conventionally fractionated EBRT involving full bladder protocols, in which rectal volume reductions were observed through the course of therapy [16, 19, 29]. It is also possible that the strict adherence to and cumulative effect of daily rectal emptying instructions involving fiber supplementation, simethicone, and enemas may partially explain the decreasing rectal volume over the treatment course. In addition, acute rectal symptoms with progression of treatment, namely tenesmus, may further propagate the rectal volume reduction observed [30]. While the literature concerning rectal displacement in SBRT patients is limited, a recent dosimetric study suggested that rectum volume receiving 75% of the prescribed dose can increase significantly during SBRT due to interfraction organ displacement [31]. As in bladder dose volume studies, prior studies of rectal displacement in relation to dose exposure involved conventionally fractionated EBRT employing more generous PTV margins and substantially greater number of fractions over several weeks and therefore may not be applicable to more precise ultra-hypofractionated treatments, especially considering the increased use of rectal spacers to mitigate rectal dose. Furthermore, while there are specific dose constraint guidelines for the rectum in conventional EBRT and brachytherapy, prospectively validated dose constraint guidelines for ultra-hypofractionated SBRT have yet to be established [32, 33].

Based on an evaluation of rectal tolerance in a Phase I/II trial, Kim et al. [34] suggested that limiting 35% rectal circumference exposure to less than 39 Gy over 5 fractions may reduce the risk for delayed rectal toxicity. Therefore, while studies have reported acceptable levels of rectal toxicity with prostate SBRT and our current assessment yielded nonexistent-to-exceedingly low rates of GI toxicities, ongoing prospective trials with long-term follow up must be evaluated before we can fully understand the dose-volume relationships and the potential impact of interfractional organ displacement [1, 5, 13, 35–38].

The key strengths of this study include its use of an objectively verified and consistent process of organ delineation, as well as its analysis of a large number of CT images. A limitation of this study was the inferior image quality produced by the CBCTs, which might have resulted in errors in organ delineation. Similarly, other artifacts due to motion and metal may have also resulted in contouring errors. As a single, trained reviewer performed the organ delineations with a high degree of consistency, any major errors were likely mitigated. Another limitation is the retrospective nature of the study and the absence of detailed toxicity outcomes to analyze in the context of our organ displacement and dosimetric data. Future prospective evaluation with long term follow up of toxicities as well as assessment of intrafractional volumetric consequences would certainly be needed to validate our hypothesis-generating observation of minimal clinical impact from strict bladder/rectal filling protocols. In terms of measuring organ displacement, an indirect measurement of center of mass changes to contoured volumes had to be employed due to limitations in geometric data generated on the treatment planning system.

Lastly, even with consistent organ delineation by a single reviewer and a large number of CT images analyzed, the post hoc nature of using clinical CBCTs invariably represents an additional limitation. Namely, the field of view (FOV) of a small number of the CBCTs were optimized for visualization of fiducial markers and pelvic bony landmarks, while minimizing superior and inferior borders to limit image acquisition time and unwarranted radiation exposure. Inevitably, the superior borders of bladders in 69 of 425 CBCTs (16.2%) throughout all fractions and 10 of 85 fraction #5 CBCTs (11.8%) were out of FOV to be precisely contoured. This was due to the FOV not extending superiorly to account for overfilled bladders at the time of image acquisition (average volumes of bladder out of CBCT FOV: 414.5 ± 116.8 mL; average volumes of bladder within CBCT FOV: 195.8 ± 105.1 mL). The degree of bladder overfilling in these CBCTs were remarkable enough to limit any major changes to the overall pattern of bladder volume decline, COM, or dosimetry to warrant exclusion of patients from analyses.

## Conclusions

Despite strict bladder filling instructions and a considerably truncated number of treatment days, a highly variable and decreasing trend in interfractional bladder and rectal volumes were observed in this SBRT cohort during the course of therapy. Such patterns in volumetric variations translated to center of mass displacement of the bladder trigone anteriorly and were associated with changes to OAR dosimetry. While statistically significant, the relatively small changes in OAR dose and displacement did not correlate with any detrimental clinical outcomes on our preliminary analyses. This study suggests that future prospective evaluation of the optimal bladder/rectal filling during prostate SBRT is necessary and perhaps less strict bladder filling and rectal emptying protocols may be required for treatment.

## Data Availability

The datasets used and/or analysed during the current study are available from the corresponding author on reasonable request.
